# Clinical role of serum histone deacetylase 4 measurement in acute ischemic stroke: Relation to disease risk, severity, and prognosis

**DOI:** 10.1002/jcla.24372

**Published:** 2022-03-30

**Authors:** Min Wang, XuYang Pang, Huaihai Lu, Xudong Wang

**Affiliations:** ^1^ Neurology Department 3 HanDan Central Hospital Handan China; ^2^ Department of Critical Care Medicine The Second Hospital of Hebei Medical University Shijiazhuang China

**Keywords:** acute ischemic stroke, cytokines, HDAC4, NIHSS score, prognosis

## Abstract

**Objective:**

Histone deacetylase 4 (HDAC4) is engaged in the pathophysiology of acute ischemic stroke (AIS) through modulating atherosclerosis, inflammation and neurocyte death. This study aimed to investigate the clinical role of HDAC4 in AIS.

**Methods:**

Serum samples were collected from 176 AIS patients and 80 controls for HDAC4 detection by enzyme‐linked immunosorbent assay (ELISA). In AIS patients, disease severity was assessed by National Institute of Health Stroke Scale (NIHSS) score and their recurrence‐free survival (RFS) and overall survival (OS) were calculated, inflammatory cytokines and adhesion molecules were detected by ELISA.

**Results:**

HDAC4 was declined in AIS patients vs. controls (*p* < 0.001), it also had certain ability of distinguishing AIS patients from controls with an area under curve of 0.748 (95% confidence interval: 0.689–0.806). Among AIS patients, HDAC4 was negatively linked with NIHSS score (*p* < 0.001) but no other clinical features (all *p* > 0.05). Moreover, HDAC4 was negatively related to interleukin (IL)‐17 (*p* = 0.010) and tumor necrosis factor alpha (*p* = 0.001), while it was not correlated with IL‐1β (*p* = 0.081) or IL‐6 (*p* = 0.074). Furthermore, HDAC4 was negatively associated with intercellular cell adhesion molecule‐1 (*p* < 0.001) and vascular cell adhesion molecule‐1 (*p* = 0.003). During a median follow‐up of 19.0 months, 17 (9.7%) patients had recurrence and 10 (5.7%) patients died. Additionally, high HDAC4 was linked with prolonged RFS (*p* = 0.044) but not OS (*p* = 0.079).

**Conclusion:**

HDAC4 possesses the potential to monitor disease risk, inflammation and estimate recurrence of AIS, while further study with larger scale is needed to verify our findings.

## Graphical abstract

HDAC4 was declined, negatively correlated with disease severity, inflammatory cytokines, and adhesion molecules, and its elevation was linked with decreased recurrence in AIS.

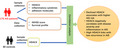

## 1 INTRODUCTION

Acute ischemic stroke (AIS) is viewed as one of main causes of neurologic death worldwide.^1^, ^2^ In China, stroke is the third highest cause of death with mortality of approximately 1.5 million in 2018,^3^ which AIS may result in permanent disability, both functionally and cognitively; meanwhile, the morbidity of AIS is continuous increasing, which causes a huge economical and humanistic burden.^4^, ^5^ Over the decades, great advances have made in the strategies for AIS management such as neuroprotective treatment, intravenous thrombolysis, and anticoagulation treatment, while AIS patients still suffer from elevating recurrence and increasing mortality.^6^, ^7^, ^8^ In view of that there still exists several challenges in the management of AIS, the identification of reliable biomarker to monitor disease risk, severity, and prognosis is crucial.

Histone deacetylase 4 (HDAC4), a crucial number of epigenetic modifier enzymes, is closely involved in various cellular processes such as apoptosis, senescence, and differentiation.^9^ Of note, accumulating researches have reported that HDAC4 is tightly involved in the pathophysiology of AIS.^10^, ^11^, ^12^, ^13^, ^14^ For instance, HDAC4 is engaged in progression of atherosclerosis *via* regulating endothelial cell apoptosis^10^; moreover, it has been proposed that HDAC4 is able to regulate vascular inflammation^11^; furthermore, HDAC4 also inhibits neuron cell death in oxygen‐glucose deprivation (OGD)‐treated mice neurons, indicating its potential of neuroprotection^12^; in addition, HDAC4 is able to inhibit adhesion molecule (VCAM‐1), which is involved in the all stage of atherosclerosis, an important risk factor in pathology of AIS.^15^, ^16^ Based on the above‐mentioned data, it could be deduced that HDAC4 might play an important clinical role in AIS, while related information is scarce.

Thus, the current study explored the association of HDAC4 with disease risk, disease severity, inflammation, adhesion molecules, and prognosis in AIS patients.

## 2 METHODS

### 2.1 Participants

This study serially enrolled 176 first‐episode AIS patients between June 2017 and June 2021. The patients with the following criteria were included in the study: (i) confirmed as AIS according to the American Stroke Association Guideline^17^; (ii) more than 18 years old; (iii) without intracranial hemorrhage based on the images of computed tomography (CT) scan or magnetic resonance angiography (MRA); (iv) volunteered to participate in the study and be followed up regularly. The patients who met the following conditions were excluded from the study: (i) unwilling to provide peripheral blood (PB) samples for study use; (ii) presented with severe infection; (iii) had a prior history of immune system disease (to eliminate the impact on the assessment of inflammatory cytokines), cancer or malignancy; (iv) pregnancy or nursing mother. In addition, this study also recruited 80 subjects from high‐risk population as controls, who had at least two high‐risk factors of stroke, including history of smoke, hypertension, hyperlipidemia, hyperuricemia, diabetes mellitus (DM), and chronic kidney disease (CKD). In order to match the demographics of controls with the included AIS patients, the recruitment of controls was started from June 2020 to June 2021. Referring to the age range and gender distribution in the AIS cohort, the age of controls was required to limited in the range of 50–80 years old, and the gender ratio of controls was limited as 3:2 (male vs. female). The controls were ineligible if they met the exclusion criteria for AIS patients or had a history of stroke. The study was admitted by the Ethics Committee. All participants or their statutory guardians signed the informed consents.

### 2.2 Collection of clinical features and samples

Clinical features (including demographics and high‐risk factors of stroke) were collected from AIS patients on the day of hospitalization as well as from controls on the day of recruiting. Besides, National Institutes of Health Stroke Scale (NIHSS) score (0–42 points) was collected from AIS patients at admission to assess the disease severity: normal (0 points), wild (1–4 points); moderate (5–15 points); moderate to severe (16–20 points); severe (21–42 points). For sample collections, PB samples were collected from AIS patients within 24 h of admission as well as from controls within 24 h of enrollment.

### 2.3 Determination of HDAC4, inflammatory cytokines and adhesion molecules

Within half an hour of after collection, PB samples were centrifuged using centrifuge at 2500 *g* revolutions per minute for 10 min to isolate serum samples, and were stored at −80℃ for further detection. Then, serum samples were used to detect the level of HDAC4, inflammatory cytokines (Interleukin [IL]‐1β, IL‐6, IL‐17A, Tumor Necrosis Factor‐α [TNF‐α]) and adhesion molecules (intercellular cell adhesion molecule‐1 (ICAM‐1) and vascular cell adhesion molecule‐1 [VCAM‐1]) by enzyme‐linked immunosorbent assay (ELISA) using commercial Human ELISA Kits. The kits for HDAC4 detection were purchased from Shanghai Enzyme‐linked Biotechnology Co., Ltd (Shanghai, China), and the kits used to assess the level of inflammatory cytokines and adhesion molecules were purchased from Bio‐Techne China Co., Ltd. (Shanghai, China). The sensitivity of the kits was as follows: HDAC4, 7.81 pg/ml; IL‐1β, 1 pg/ml; IL‐6, 0.7 pg/ml; IL‐17A, 15 pg/ml; TNF‐α, 6.23 pg/ml; ICAM‐1, 0.254 ng/ml; VCAM‐1, 1.26 ng/ml. All experiments were carried out in stringent accordance with the protocols provided by the manufacturer.

### 2.4 Follow‐up

AIS patients were followed up regularly until September 30, 2021, and the disease status during follow‐up was recoded. Then, the follow‐up data (from June 2017 to September 30, 2021) were used to calculate recurrence‐free survival (RFS) and overall survival (OS). RFS was defined as the time interval from admission to disease recurrence or patient's death, and OS was defined as the time interval from admission to patient's death.

### 2.5 Statistical analysis

Statistical analysis and graph construction were, respectively, completed using SPSS V.24.0 (IBM Corp., Armonk, New York, USA) and GraphPad Prism V.6.01 (GraphPad Software Inc., San Diego, CA, USA). Difference of HDAC4 expression between AIS patients and controls were compared using the Mann–Whitney U‐test, and receiver operating characteristic (ROC) curve was applied to estimate the ability of HDAC4 expression in identifying different subjects. Correlation of two variables was assessed using the Mann–Whitney U‐test or Spearman's rank correlation test. RFS and OS were described using Kaplan–Meier curves and determined by log‐rank test. *p*‐value < 0.05 was considered statistically significant.

## 3 RESULTS

### 3.1 Clinical features in AIS patients and controls

Among 80 controls, the mean age was 63.7 ± 8.0 years; meanwhile, there were 32 (40.0%) females and 48 (60.0%) males. Furthermore, 176 AIS patients showed with a mean age of 64.0 ± 9.4 years, consisting of 52 (29.5%) females and 124 (70.5%) males. Generally, demographic characteristics and most high‐risk factors of stroke were of no difference in AIS patients vs. controls (all *p* > 0.05), only the percentage of AIS patients with hyperuricemia was higher compared to the percentage of controls with hyperuricemia (47.7% vs. 22.5%, *p* < 0.001). In AIS patients, the mean NIHSS score was 8.4 ± 5.0; regarding inflammatory cytokines, the median values (interquartile range (IQR)) of interleukin (IL)‐1β, IL‐6, IL‐17 and tumor necrosis factor alpha (TNF‐α) were 2.1 (1.2–2.9) pg/ml, 15.1 (11.2–21.8) pg/ml, 39.7 (30.4–53.8) pg/ml and 132.6 (93.3–190.1) pg/ml, respectively; as for adhesion molecules, the median values (IQR) of intercellular cell adhesion molecule‐1 (ICAM‐1) and vascular cell adhesion molecule‐1 (VCAM‐1) were 88.5 (51.8–140.3) ng/ml and 567.1 (452.2–793.7) ng/ml, accordingly (Table 1).

**TABLE 1:** Clinical features

Items	Controls (*N* = 80)	AIS patients (*N* = 176)	*p*‐value
Demographics			
Age (years), mean ± SD	63.7 ± 8.0	64.0 ± 9.4	0.791
Gender, No. (%)			
Female	32 (40.0)	52 (29.5)	0.099
Male	48 (60.0)	124 (70.5)	
BMI (kg/m^2^), mean ± SD	23.8 ± 3.2	23.9 ± 2.8	0.854
High‐risk factors of stroke			
Current smoke, No. (%)			
No	48 (60.0)	92 (52.3)	0.250
Yes	32 (40.0)	84 (47.7)	
Hypertension, No. (%)			
No	17 (21.3)	33 (18.8)	0.640
Yes	63 (78.7)	143 (81.2)	
Hyperlipidemia, No. (%)			
No	38 (47.5)	89 (50.6)	0.649
Yes	42 (52.5)	87 (49.4)	
Hyperuricemia, No. (%)			
No	62 (77.5)	92 (52.3)	<0.001
Yes	18 (22.5)	84 (47.7)	
Diabetes mellitus, No. (%)			
No	67 (83.7)	133 (75.6)	0.142
Yes	13 (16.3)	43 (24.4)	
CKD, No. (%)			
No	66 (82.5)	137 (77.8)	0.394
Yes	14 (17.5)	39 (22.2)	
NIHSS score, mean ± SD	–	8.4 ± 5.0	–
Inflammatory cytokines			
IL‐1β (pg/ml), median (IQR)	–	2.1 (1.2–2.9)	–
IL‐6 (pg/ml), median (IQR)	–	15.1 (11.2–21.8)	–
IL‐17 (pg/ml), median (IQR)	–	39.7 (30.4–53.8)	–
TNF‐α (pg/ml), median (IQR)	–	132.6 (93.3–190.1)	–
Adhesion molecules			
ICAM‐1 (ng/ml), median (IQR)	–	88.5 (51.8–140.3)	–
VCAM‐1 (ng/ml), median (IQR)	–	567.1 (452.2–793.7)	–

Abbreviations: AIS, acute ischemic stroke; BMI, body mass index; CKD, chronic kidney disease; ICAM‐1, intercellular cell adhesion molecule‐1; IL‐17, interleukin 17; IL‐1β, interleukin‐1β; IL‐6, interleukin‐6; IQR, interquartile range; NIHSS, National Institute Health of Stroke Scale; SD, standard deviation; TNF‐α, tumor necrosis factor alpha; VCAM‐1, vascular cell adhesion molecule‐1.

### 3.2 HDAC4 in AIS patients and controls

**FIGURE 1:**
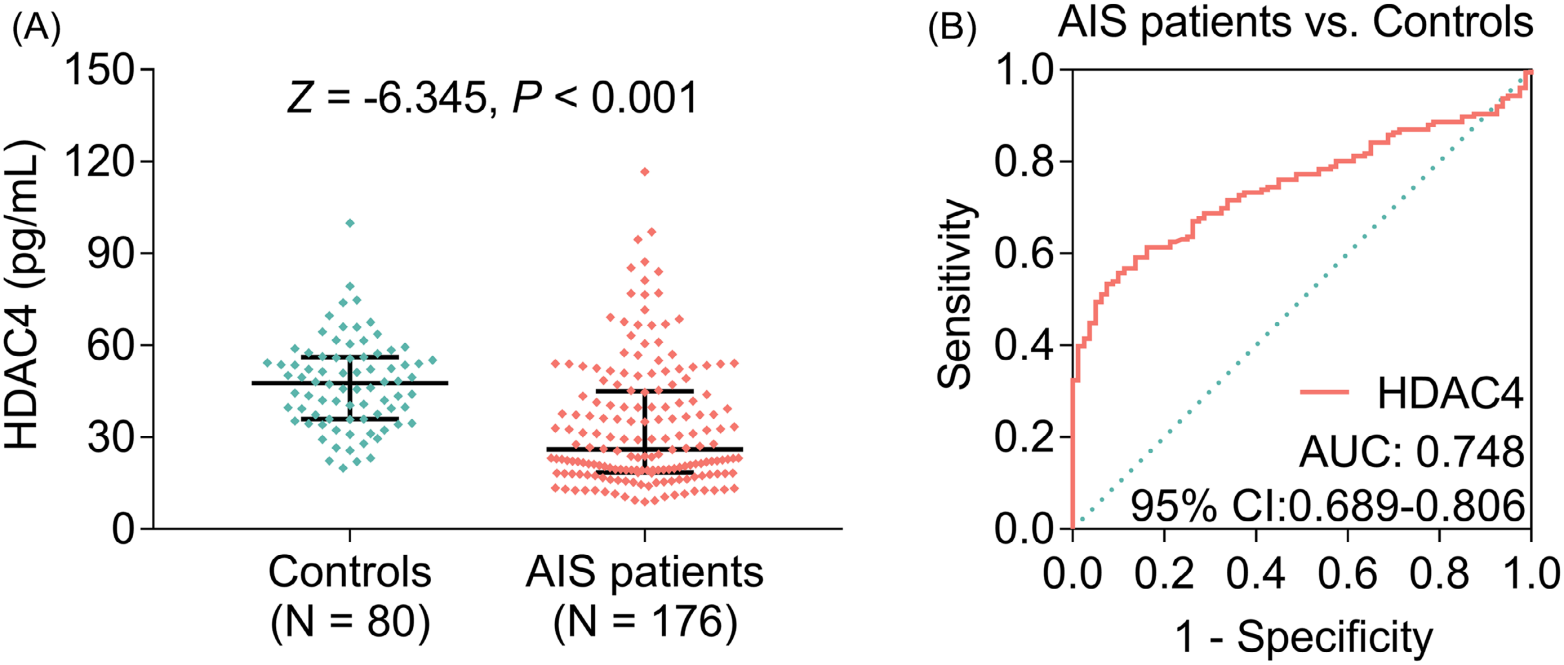
Comparison of HDAC4 between AIS patients and controls. Comparison of HDAC4 between AIS patients and controls (A); the ability of HDAC4 to discriminate AIS patients from controls (B)

HDAC4 was declined in AIS patients vs. controls (median (IQR): 26.1 (18.4–45.1) pg/ml vs. 47.7 (35.9–56.2) pg/ml, *p* < 0.001) (Figure 1A). Further ROC curve presented that HDAC4 had certain ability of discriminating AIS patients from controls, with an area under curve (AUC) of 0.748 (95% confidence interval (CI): 0.689–0.806); meanwhile, HDAC4 expression was 27.85 at the best cut‐off point (the point with maximum value of the sum of sensitivity and specificity); the sensitivity and specificity were 0.534 and 0.925 at the best cut‐off point, respectively (Figure 1B).

### 3.3 The correlation of HDAC4 with disease characteristics, inflammatory cytokines, and adhesion molecules among AIS patients

**FIGURE 2:**
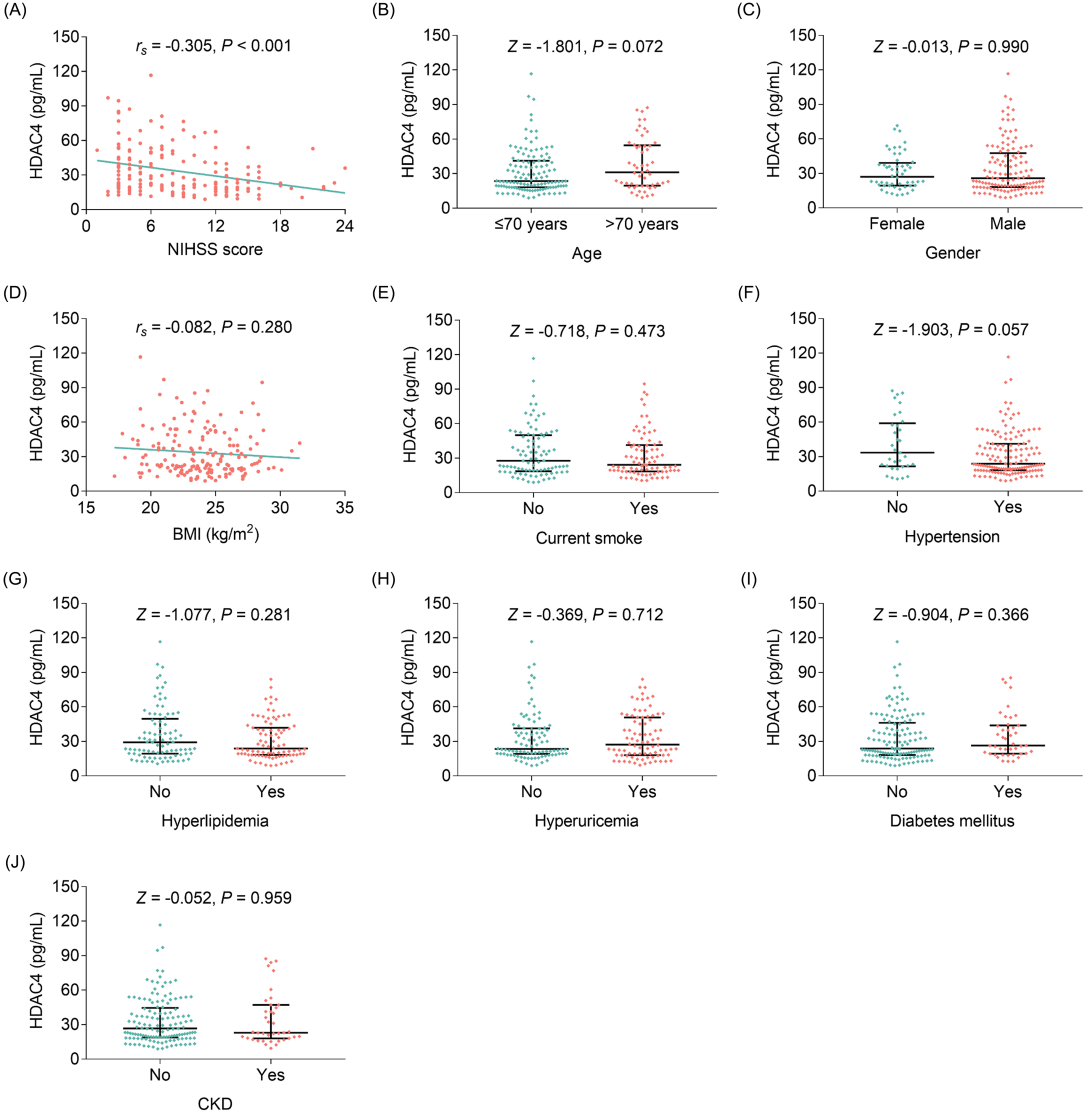
Correlation of HDAC4 with clinical features in AIS patients. Correlation of HDAC4 with NIHSS score (A), age (B), gender (C), BMI (D), current smoke (E), hypertension (F), hyperlipidemia (G), hyperuricemia (H), diabetes mellitus (I), and CKD (J)

**FIGURE 3:**
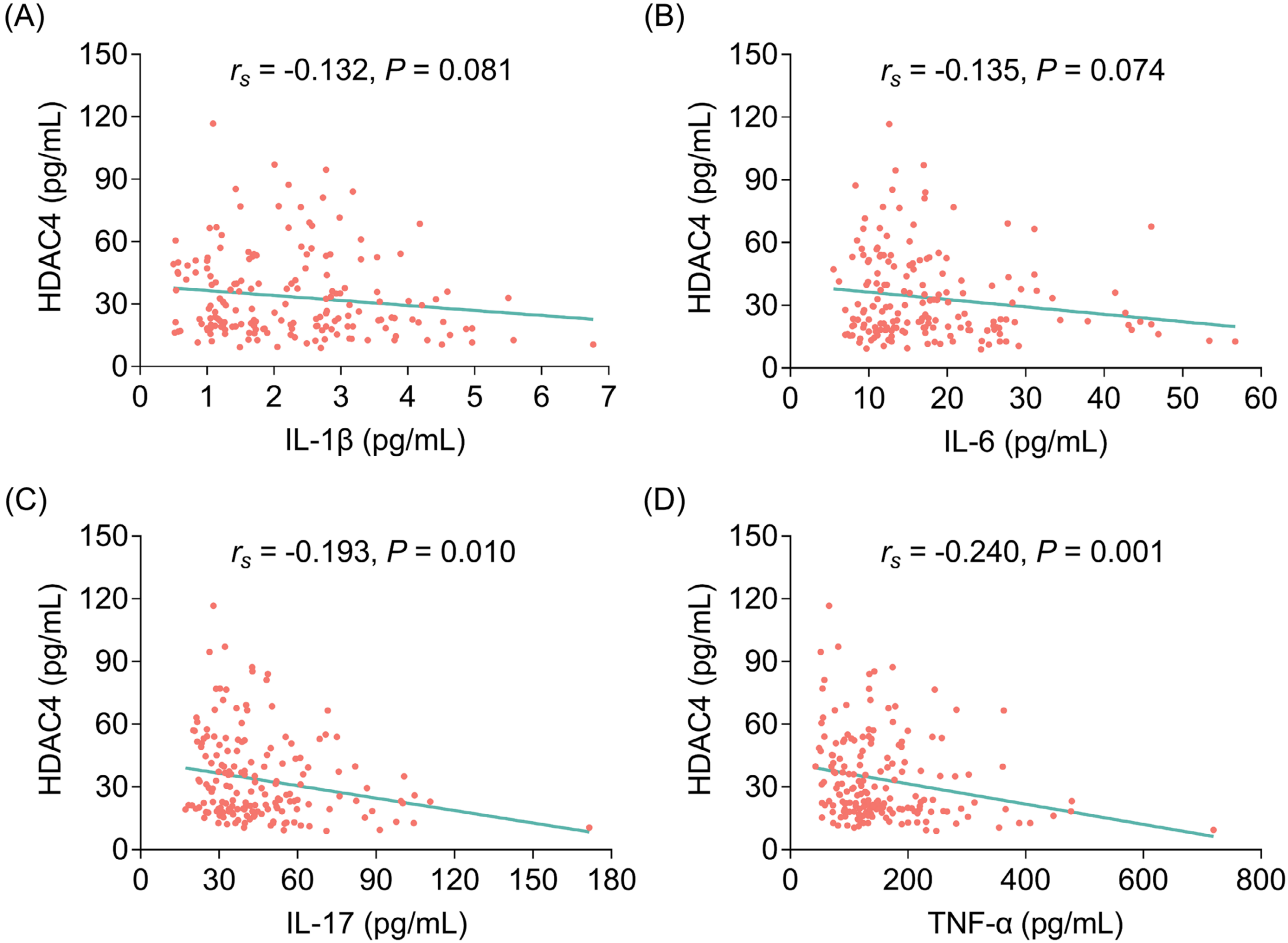
Correlation of HDAC4 with inflammatory cytokines. Correlation of HDAC4 with IL‐1β (A), IL‐6 (B), IL‐17 (C), and TNF‐α (D)

**FIGURE 4:**
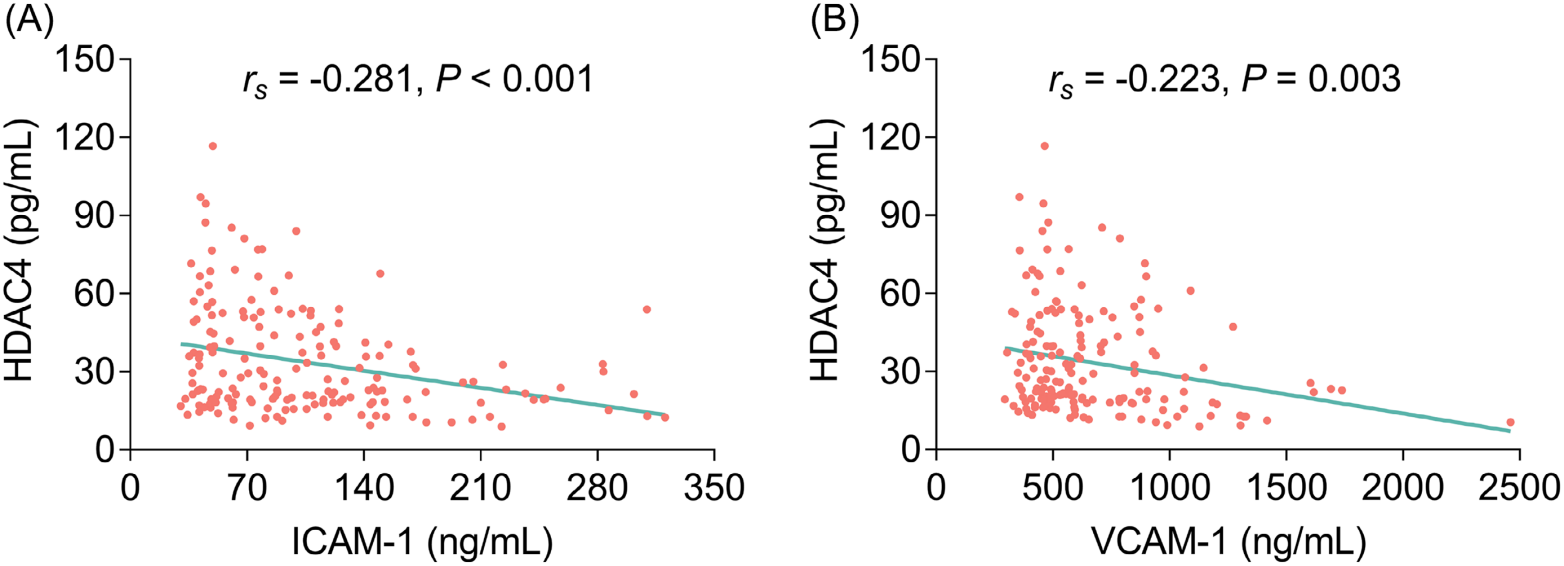
Correlation of HDAC4 with adhesion molecules. Correlation of HDAC4 with ICAM‐1 (A) and VCAM‐1 (B)

HDAC4 was negatively correlated with NIHSS score (*p* < 0.001); while no correlation was found in HDAC4 with demographic characteristics or high‐risk factors of stroke among AIS patients (all *p* > 0.05) (Figure 2A‐J). Besides, HDAC4 presented a declined trend along with elevated disease severity grade by NIHSS score (*p* < 0.001) (Figure S1). Regarding the correlation between HDAC4 and inflammatory cytokines, HDAC4 was negatively correlated with IL‐17 (*r_s_* = −0.193, *p* = 0.010) and TNF‐α (*r_s_* = −0.240, *p* = 0.001); however, HDAC4 was not correlated with IL‐1β (*r_s_* = −0.132, *p* = 0.081) or IL‐6 (*r_s_* = −0.135, *p* = 0.074) (Figure 3A‐D). In terms for the association between HDAC4 and adhesion molecules, HDAC4 was negatively correlated with ICAM‐1 (*r_s_* = −0.281, *p* < 0.001) and VCAM‐1 (*r_s_* = −0.223, *p* = 0.003) (Figure 4A,B).

### 3.4 The association of HDAC4 with prognosis among AIS patients

**FIGURE 5:**
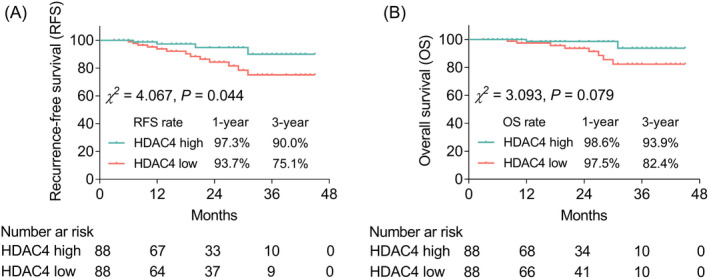
Correlation of HDAC4 with AIS outcome. Correlation of HDAC4 with RFS (A) and OS (B)

During a median follow‐up of 19.0 months, disease recurred in 17 (9.7%) patients, and 10 (5.7%) patients died. Furthermore, AIS patients were classified as patients with HDAC4 high and patients with HDAC4 low based on the median level of HDAC4 in AIS patients (26.1 pg/ml). Additionally, 1‐year and 3‐year RFS rates were 97.3% and 90.0%, respectively, in patients with HDAC4 high, and they were 93.7% and 75.1%, accordingly in patients with HDAC4 low; furthermore, HDAC4 high expression was correlated with prolonged RFS (*p* = 0.044) (Figure 5A). In addition, 1‐year and 3‐year OS rates were 98.6% and 93.9%, respectively, in patients with HDAC4 high, and they were 97.5% and 82.4%, accordingly in patients with HDAC4 low; moreover, HDAC4 high expression was not correlated with OS (*p* = 0.079) (Figure 5B).

## 4 DISCUSSION

It has been presented that HDAC4 is declined in ODG‐treated PC12 cells and ischemic stroke model mice.^18^ However, the expression and the clinical role of HDAC4 in AIS patients is still unclear. In the current study, we found that HDAC4 was generally decreased in AIS patients compared with controls, although some AIS patients exhibited extremely high HDAC4; meanwhile, declined HDAC4 predicted higher AIS risk. The potential explanations might be that: (1) declined HDAC4 could promote the progression of atherosclerosis, which consequently resulted in the occurrence of AIS^10^; (2) decreased HDAC4 might promote arterial stenosis *via* promoting vascular endothelial cell apoptosis, which was involved in the pathogenesis of AIS.^1^, ^10^, ^19^ Apart from that, negative association was also found between HDAC4 and disease severity reflected by NIHSS score, which might be caused by that: (1) HDAC4 could alleviate neuron injury through inhibiting neuron cell apoptosis, which suppressed disease severity of AIS^12^; (2) HDAC4 could alleviate the atherosclerosis lesions *via* inhibiting vascular endothelial cell damage, consequently decreasing AIS severity.^10^

The data about the association of HDAC4 with inflammation in AIS patients is obscured. To solve this issue, we detected several inflammatory cytokines in AIS patients and explored their association with HDAC4 in the current study, which presented that HDAC4 was negatively correlated with inflammation to some extent. The potential reason might be that HDAC4 might inhibit inflammation through several pathways, such as mitogen‐activated protein kinase and nuclear factor kappa‐B signaling pathways.^20^, ^21^, ^22^, ^23^ Furthermore, ICAM‐1 and VCAM‐1 are involved in the all stage of atherosclerosis, an important risk factor in pathology of AIS.^15^ Hence, we detected the level of ICAM‐1 and VCAM‐1 in AIS patients, which showed that negative correlation was found between HDAC4 and these adhesion molecules. The potential explanation might be ICAM‐1 and VCAM‐1 could regulate atherosclerosis, hence ICAM‐1 and VCAM‐1 levels could reflect the development of atherosclerosis to some extent^15^; meanwhile HDAC4 could regulate the invasion of vascular smooth muscle cells and apoptosis of endothelial cells to inhibit the development of atherosclerosis,^10^, ^24^ thus, HDAC4 was negatively correlated with these two adhesion molecules.

Until now, recurrence is viewed as a huge challenge for AIS, which causes prolonged hospitalization and elevated mortality.^25^ Therefore, the exploration of potential biomarker to predict recurrence is urgent among AIS patients. Surprisingly, in the current study, we found that elevated HDAC4 was correlated with higher RFS but not OS. The possible explanations might be that: (1) elevated HDAC4 could inhibit the disease severity and progression of AIS through suppressing inflammation and restraining the development of atherosclerosis (above‐mentioned), which led to better RFS; (2) the events of mortality was relatively low in the current study, thus it might be difficult to observe statistical significance in the correlation between HDAC4 and OS.

There were several limitations in the current study: (1) we did not have a tailed protocol for current study to conduct a regular and stable follow‐up, and the follow‐up in our study was based on routine schedule and the intention of patients, which might result in high number of patients who lost follow‐up; hence, the patients who lost follow‐up were treated as censored data (2) the role of HDAC4 in regulatory mechanism of AIS could be explored; (3) the enrolled patients were first‐episode AIS patients, hence the clinical role of HDAC4 in recurrent AIS could be further explored; (4) HDAC4 participated in stroke‐induced angiogenesis, which was a protective process after stroke^14^; thus, the correlation of HDAC4 with markers of angiogenesis, such as VEGF, HIF‐1 alpha could be explored in the further study; (5) a huge number of patients lost follow‐up in the current study; (6) lacking of transcriptive level of HDAC4 detection in the present study.

To be conclusive, HDAC4 possesses the potential to monitor disease risk, inflammation and estimate recurrence of AIS, while further study with larger scale is needed to verify our findings.

## CONFLICT OF INTEREST

The authors declare they have no conflict of interests.

## Supporting information

Fig S1

## Data Availability

Data sharing is not applicable to this article as no datasets were generated or analyzed during the current study.
