# Supplementary figures and images for: Clinical role of serum histone deacetylase 4 measurement in acute ischemic stroke: Relation to disease risk, severity, and prognosis
Source: J Clin Lab Anal. 2022 Mar 30;36(5):e24372. doi: 10.1002/jcla.24372 (PMC9102630; doi:10.1002/jcla.24372)

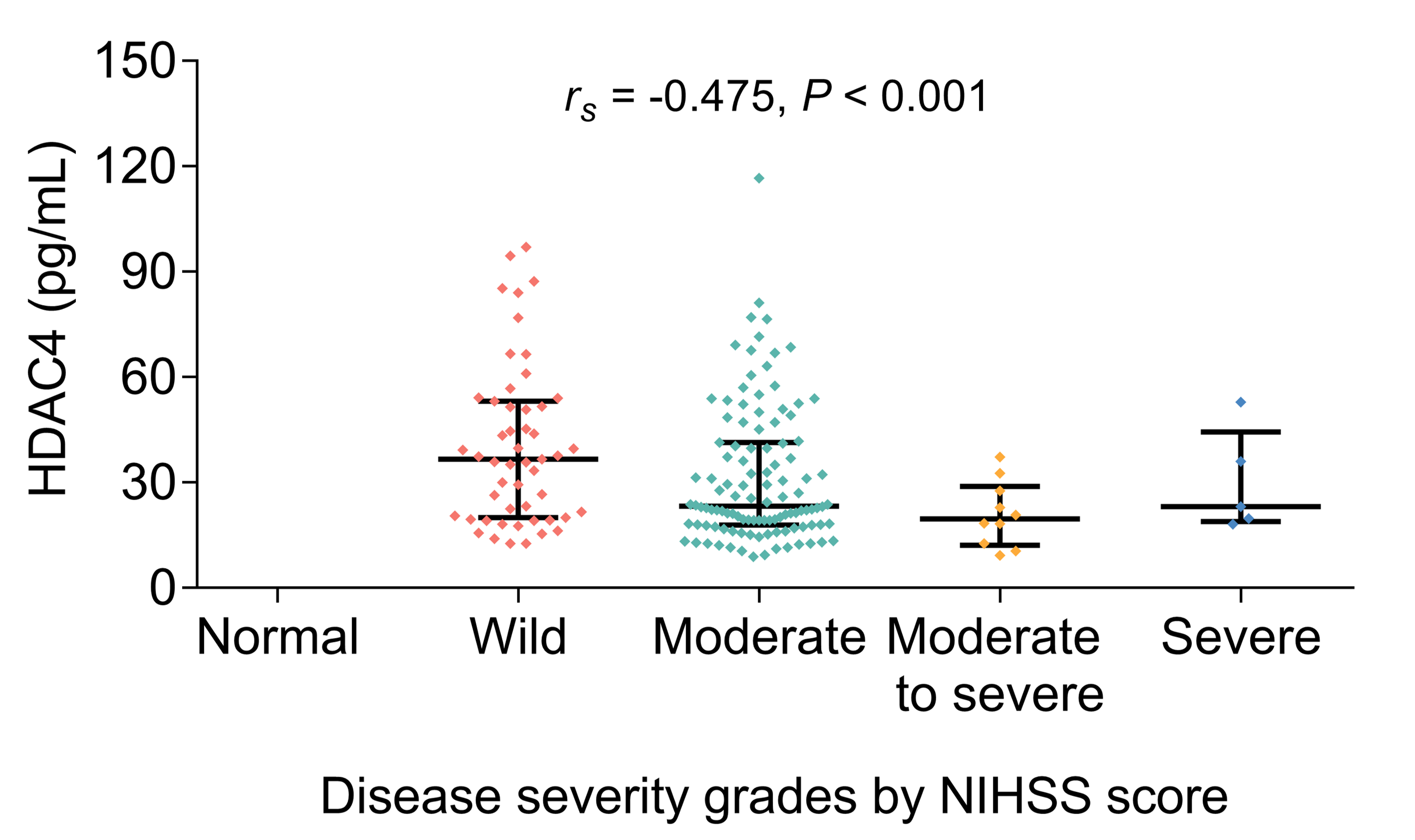

Supplement: Supplementary file 1 — Fig S1 [file JCLA-36-e24372-s001.tif]
